# The Buffalo Clinical Society

**Published:** 1891-12

**Authors:** 


### Society Meetings.

The Buffalo Clinical Society held its first meeting, for organization, Saturday evening, November 21, 1891, at the Niagara Medical College building, on Ellicott street. The officers are: President, Dr. John H. Pryor; Vice-President, Dr. Eugene H. Smith; Secretary and Treasurer, Dr. M. V. Ball; Councilmen: (three years) Dr. A. A. Hubbell, (two years) Dr. W. H. Heath, (one year) Dr. H. M. Mickle. Meetings will be held on the first Saturday in each month, at the Niagara Medical College building.

